# Identification of a better *Homo sapiens *Class II HDAC inhibitor through binding energy calculations and descriptor analysis

**DOI:** 10.1186/1471-2105-11-S7-S16

**Published:** 2010-10-15

**Authors:** Usman Sumo Friend Tambunan, Evi Kristin Wulandari

**Affiliations:** 1 Laboratory of Bioinformatics, Department of Chemistry, Faculty of Mathematics and Natural Science, University of Indonesia, Depok 16424, Indonesia

## Abstract

Human papillomaviruses (HPVs) are the most common on sexually transmitted viruses in the world. HPVs are responsible for a large spectrum of deseases, both benign and malignant. The certain types of HPV are involved in the development of cervical cancer. In attemps to find additional drugs in the treatment of cervical cancer, inhibitors of the histone deacetylases (HDAC) have received much attention due to their low cytotoxic profiles and the E6/E7 oncogene function of human papilomavirus can be completely by passed by HDAC inhibition. The histone deacetylase inhibitors can induce growth arrest, differentiation and apoptosis of cancer cells. HDAC class I and class II are considered the main targets for cancer. Therefore, the six HDACs class II was modeled and about two inhibitors (SAHA and TSA) were docked using AutoDock4.2, to each of the inhibitor in order to identify the pharmacological properties. Based on the results of docking, SAHA and TSA were able to bind with zinc ion in HDACs models as a drug target. SAHA was satisfied almost all the properties i.e., binding affinity, the Drug-Likeness value and Drug Score with 70% oral bioavailability and the carbonyl group of these compound fits well into the active site of the target where the zinc is present. Hence, SAHA could be developed as potential inhibitors of class II HDACs and valuable cervical cancer drug candidate.

## Introduction

The human papillomavirus (HPV) is a family of sexually transmitted, double-stranded DNA viruses with over 100 different genotypes identified till date. It is associated with many different types of cancers including cervical, vaginal, head and neck, penile and anal cancer. With approximately 450,000 newly diagnosed cases each year and a 50% mortality rate [1,2], cervical cancer is the second most common cause of cancer-related death in women worldwide and it is almost always associated with HPV [3,4]. Cervical cancer is the most common cancer of women in most developing countries, where it may account for as many as one fourth of female cancers [5].

HPV genotypes are divided into the low risk and high risk categories based on the spectrum of lesions they induce. The low-risk types induce only benign genital warts and include HPV 6 and 11. The high-risk group containing HPV 16, 18, 31, 33, 45 and 56 is associated with the development of anogenital cancers and can be detected in 99% of cervical cancers [6], with HPV16 found in 50% of cases [7].

A consistently effective and safe treatment for HPV infections is currently not yet available. Present therapeutic options are more directed at surgical eradication and/or by destroying malignant lesions via physical or chemotherapeutical intervention. A majority of these treatments have been developed empirically, few have been thoroughly tested, but none of them are completely satisfactory. In attemps to find additional drugs in the treatment of cervical cancer, inhibitors of the histone deacetylases have received much attention due to their low cytotoxic profiles [5].

The structural modification of histones is regulated mainly by acetylation/deacetylation of the N-terminal tail and is crucial in modulating gene expression, because it affects the interaction of DNA with transcription-regulatory non-nucleosomal protein complexes. The balance between the acetylated/deacetylated states of histones is mediated by two different sets of enzymes: histone acetyltransferases (HATs) and histone deacetylases (HDACs). HATs prefentially acetylate specific lysine substrates among other nonhistones protein substrates and transcription factors, affecting DNA-binding properties and, in turn, altering gene transcription. HDACs restore the positive charge on lysine residues by removing acetyl groups and thus are involved primarily in the repression of gene transcription by compacting chromatin structure. Therefore, open lysine residues attach firmly to the phosphate backbone of the DNA, preventing transcription. In this tight conformation, transcription factors, regulatory complexes, and RNA polymerases cannot bind to DNA Acetylation relaxes the DNA conformation, making it accessible to transcription machinery. High levels of acetylation of core histones are seen in chromatin-containing genes, which are highly transcribed genes; genes that are silent are associated with low levels of acetylation. Inappropriate silencing of critical genes can result in one or both hits of tumor suppressor gene inactivation in cancer [8].

Members of the classical HDAC family fall into two different phylogenetic classes, namely class I and class II. The class I HDACs (HDAC1, HDAC2, HDAC3, and HDAC8) are most closely related to the yeast (*Saccharomyces cerevisiae*). Class II HDACs (HDAC4, HDAC5, HDAC6, HDAC7, HDAC9, and HDAC10) share domains with similarity to HDA1, another deacetylase found in yeast [9].

The inhibition of HDAC activity by a specific inhibitor induces growth arrest, differentiation, and apoptosis of transformed cells as well as several cancer cells [10]. Recent studies were directed to investigate the molecular effects of HDAC inhibition on cervical carcinoma cells as well as on primary human foreskin keratinocytes, separately immortalized with amphotropic retroviruses that carry the open reading frames of HPV 16 E6, E7 or E6/E7.

In these experiments one could show that E6/E7 oncogene function of human papillomavirus can be completely bypassed by HDAC inhibition. Both malignant and immortalized HPV 16/18-positive cells became blocked in G1/S transition despite ongoing viral gene expression. G1 arrest was accompanied by a down-regulation of cyclin D and cyclin A and a concomitant up-regulation of the cyclin kinase inhibitors (CKI) *p21 *and *p27*. Binding of both CKIs led to a complete block of the cyclin-dependent kinase (cdk2) activity and in turn prevented binding of E7. This was intriguing with respect to the reversibility of HPV transformation process, since it is thought that the abrogation of the growth inhibitory function of *p21 *and *p27 *through E7 represents a key event in HPV-induced carcinogenesis. HDAC inhibitors also trigger *pRb *degradation, while *E2F *expression remained unaffected. pRb degradation is an E7- specific phenomenon, since in E6-positive cells pRb only became hypophosphorylated. The presence of *E2F *under cell cycle arrest led to a classical "conflict situation" which finally induced apoptosis [11-13]. Hence, the knowledge how the transforming potential of HPV can be bypassed without switching off viral transcription could open new therapeutical perspectives for the treatment of cervical cancer [14].

The aim of this work is to analyze the interaction of *Homo sapiens *class II HDACs with SAHA and TSA that are already in the phase I/II clinical trials based on their binding affinity and pharmacological properties. Since, no theoretical works have been carried out in identifying the properties and specificity, we intend to identify the group that could act as potential binding inhibitors.

In this paper, we present homology models of six *Homo sapiens *Class II HDACs (HDAC4, HDAC5, HDAC6, HDAC7, HDAC9, and HDAC10) that are validated by comparison with the X-ray structure of HDAC4 and HDAC7, which became available during the course of our study. Two HDAC inhibitors (SAHA and TSA) are docked to the six homology models. The pharmacological properties of SAHA and TSA were identified using Molinspiration, Osiris Property, ToxBoxes, and Toxmatch-v1.06. Therefore, the molecular binding interactions between the histone deacetylases with SAHA and TSA were analyzed to provide some insights into the molecular interactions and designing new HDAC inhibitors.

## Materials and methods

### Sequence alignment and homology modeling

Sequences of HDAC4 (Genbank Accession Number NP_006028.2,410aa), HDAC5 (Genbank Accession Number NP_005465.2,383aa), HDAC6 (Genbank Accession Number NP_006035.2,264aa), HDAC7 (Genbank Accession Number NP_056216.2,422aa), HDAC9 (Genbank Accession Number NP_848510.1,376aa), and HDAC10 (Genbank Accession Number NP_114408.3,382aa) were extracted from the NCBI protein sequence database. The eleven three-dimensional structures of HDAC4 and HDAC7 (PDB code 2H8N, 2094, 2VQJ, 2VQM, 2VQO, 2VQQ, 2VQV, 2VQW, 3C0Y, 3C0Z, and 3C10) were used to find the catalytic domain of *Homo sapiens *Class II HDACs. All sequences were imported into the ClustalW program [15] and the sequence alignment editor, BioEdit, for multiple pairwise alignments. The resulting alignments were examined manually. The four dimensional structure of HDAC (PDB code: 1C3P, 1ZZ1, 2VQM, and 3EW8) was used as a template for *Homo sapiens *Class II HDACs homology modeling in the Modeller9v7 program [16]. The multiple alignment between catalytic domain of class II HDACs and templates, using HHpred web server http://toolkit.tuebingen.mpg.de/hhpred to get an alignment pir format as an input format for homology modeling in the Modeller9v7 program [17].

### Molecular docking

**Figure 1:**
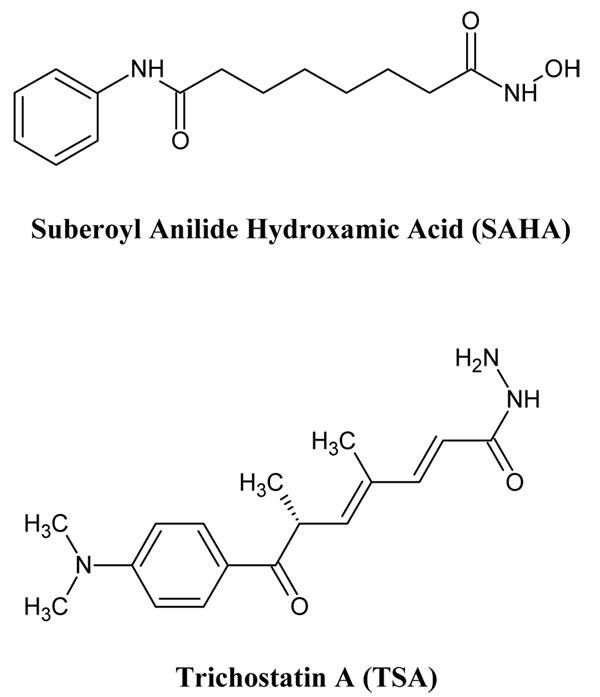
**2D Structure of SAHA and TSA.** SAHA and TSA are hydroxamic acid derivatives that can be HDAV inhibitors.

AutoDock4.2 was used for all docking calculations. The grid boxes size was implemented for HDAC4, HDAC5, HDAC6, HDAC7, HDAC9, and HDAC10 were 19.228 × -6.296 × 0.177; 19.969 × -6.093 × 0.513; 15.326 × -7.608 × 8.495; 18.484 × -6.198 × -0.744; 18.078 × -7.314 × -2.129; 17.713 × -6.707 × 1.288 points with a spacing 0.375, respectively. For SAHA and TSA, the single bonds except the amide bond were treated as active torsional bonds. One hundred docked structures, i.e. 100 runs, were generated by using genetic algorithm searches. A default protocol was applied, with an initial population of 150 randomly placed individual structures, a maximum number of 2.5 × 10^6 ^energy evaluations, and a maximum number of 2.7 × 10^4 ^generations. A mutation rate of 0.02 and a crossover rate of 0.8 were used (Figure 1).

### Binding energy calculations

The Adaptive Poisson-Boltzmann Solver (APBS) software was used for binding energy calculations [18]. Starting from the ligand conformations which is binding to Zn^2+ ^ion in class II HDACs structure obtained by the docking process, were used as an input for binding energy calculations. The pdb format of class II HDACs and ligands complex was converted to pqr format by adding missing atoms, optimizing hydrogen bonding and assigning atomic charge and radius parameters. The CHARMM22 force field parameters were assigned to the class II HDACs. For ligand parameterization requires a MOL2-format representation of the ligand to provide the necessary bonding information. In this research, MOL2-format files were obtained through PRODRG web server http://davapc1.bioch.dundee.ac.uk/prodrg/.

### Molecular descriptors calculation

Quantitative structure-activity relationships (QSARs) correlate the response with molecular properties of compounds under interest. Any compound to be considered as a lead must possess acceptable scores for all of the descriptors. Molinspiration http://www.molinspiration.com/ and Osiris Property explorer http://www.organic-chemistry.org/prog/peo/ were used to calculate twelve descriptors- logP, solubility, drug likeliness, polar surface area, molecular weight, number of atoms, number of rotatable bonds, volume, drug score and number of violations to Lipinski's rule for SAHA and TSA taken for the analysis [19].

### Toxicity prediction

Toxicity is defined as a dose-linked unsafe effect of a chemical compound on a target life form. Safety issues and ADME (Absorption, Distribution, Metabolism and Excretion) are major factors in drug failure and they are crucial to identify early in the discovery process to reduce late-stage attrition. ADME-Tox Box of PharmaAlgorithms http://pharma-algorithms.com/physchem.html was used to predict the various toxicity effects such as rat LD_50_, mouse LD_50_, oral bioavailability, Ames test, pKa and ion fraction values.

## Results and discussion

### Homology models validation

**Figure 2:**
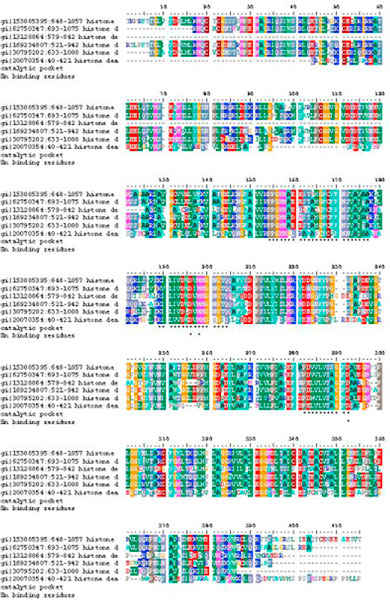
**Sequence alignment of *Homo sapiens *Class II HDACs (HDAC4, HDAC5, HDAC6, HDAC7, HDAC9, and HDAC10)**. Catalytic pocket, Zn^2+^-binding residues are marked as star.

The final result of multiple sequence alignment of the class II HDACs is shown in Figure 2. HDAC4, HDAC5, HDAC6, HDAC7, HDAC9, and HDAC10 share the same catalytic pocket and zinc binding residues. Visualization of those residues using PyMOL, showed that area was solvent accessible and has negative charge electrostatic potential surface.

After sequence alignment of the *Homo sapiens *class II HDACs, five models for each of the six HDACs were generated through the Modeller9v7 program. The quality of the models thus obtained was then evaluated with respect to the conformation of the peptide backbone and the packing environment. Generation of the Ramachandran plots using VADAR (Volume, Area, Dihedral Angel Reporter) [20] gave no residues having Phi/Psi angles in the disallowed ranges, and the percentage of residues having Phi/Psi angles in the most favorable ranges is around 97-98%, similar to the template structures used. The quality of Ramachandran plots was satisfactory for all of the models. In the second test, the stereo/packing for residues of the same type in high quality experimental structures deposited in the Protein Data Bank were compared with our HDACs models using VADAR. A score of < 5.0 or worse usually indicate poor packing. In general, our HDACs models have similar packing scores compared to the template X-ray structure. A few residues are poorly packed in these models based upon scores less than 5, but those residues are not binding ligand domain. In summary, the quality of our HDACs models has been checked by two different criteria. The results showed that our models are reliable for performing further docking studies.

### Comparison of the homology model and X-ray structure of HDAC4

During our homology modeling studies of HDACs, the X-ray structures of HDAC (PDB ID 1C3P, 1ZZ1, 2VQM, and 3EW8) cocrystallized with several inhibitors were reported at 1.80 Å, 1.57 Å, 1.80 Å, 1.80 Å resolution, respectively. Comparison of the X-ray structures to our homology model of *Homo sapiens *class II HDACs was an independent way of validating our result. With a backbone RMSD ≤ 0.5 Å for residues 648-1057 of HDAC4, 693-1075 of HDAC5, 579-842 of HDAC6, 521-942 of HDAC7, 633-1008 of HDAC9, and 40-421 of HDAC10, the *Homo sapiens *class II HDACs homology model is very similar to the X-ray structure 2VQM. The overall secondary and tertiary structures are very similar for the model and the X-ray structures.

### Docking of SAHA and TSA to HDACs models

**Figure 3:**
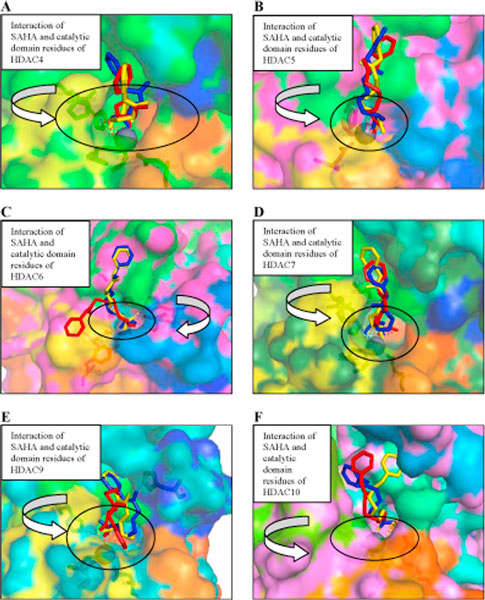
**Structures of docked SAHA with *Homo sapiens *Class II HDACs**. Three Conformations of Structures of docked SAHA with **(A) **HDAC4, **(B) **HDAC5, **(C) **HDAC6, **(D) **HDAC7, **(E) **HDAC9 and **(F) **HDAC10. A surface representation of catalytic domain of *Homo sapiens *Class II HDACs bound to SAHA. The zinc ion is shown as gray sphere. SAHA are shown as stick models colored as per docked type: red, blue, and yellow. Amino acids coordinating the zinc and forming the trihedrally coordinates are shown as sticks. Some catalytic domain of *Homo sapiens *Class II HDACs residues interacting with the docked SAHA are shown as stick models. In HDAC4-SAHA, HDAC5-SAHA, HDAC6-SAHA and HDAC7-SAHA complexes, SAHA binds the catalytic zinc ion in a bidentate fashion, with its carbonyl and hydroxyl bound to catalytic zinc ion. Whereas, in HDAC9-SAHA and HDAC10-SAHA complexes bind the catalytic zinc ion in a monodentate fashion, with its carbonyl bound to catalytic zinc ion.

**Figure 4:**
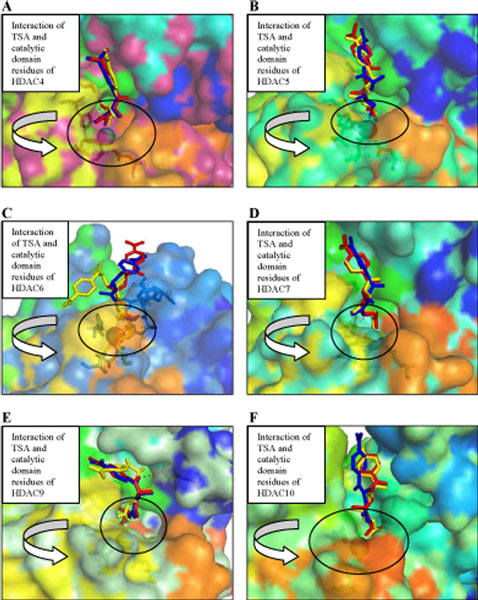
**Structures of docked TSA with HDAC Class II *Homo sapiens***. Three Conformations of Structures of docked TSA with **(A) **HDAC4, **(B) **HDAC5, **(C) **HDAC6, **(D) **HDAC7, **(E) **HDAC9 and **(F) **HDAC10. A surface representation of catalytic domain of *Homo sapiens *Class II HDACs bound to TSA. The zinc ion is shown as gray sphere. TSA are shown as stick models colored as per docked type: red, blue, and yellow. Amino acids coordinating the zinc and forming the trihedrally coordinates are shown as sticks. Some catalytic domain of *Homo sapiens *Class II HDACs residues interacting with the docked TSA are shown as stick models. Except HDAC5-TSA complex, all of *Homo sapiens *class II HDACs-TSA complexes bind the catalytic zinc ion in a monodentate fashion.

To investigate the interaction between *Homo sapiens *class II HDAC with inhibitors, we docked SAHA and TSA to the six homology models. The results for SAHA and TSA are shown in Figures 3 and 4 while the calculated binding energies are summarized in Table 1. As expected, SAHA and TSA bind in the active site of HDAC4, HDAC5, HDAC6, HDAC7, HDAC9, and HDAC10 with a similar pattern. All docked SAHA and TSA structures have the same orientation as in the X-ray structure of the HDAC4-hydroxamic acid cocrystal. In HDAC4-SAHA and HDAC4-TSA complexes, the catalytic zinc trihedrally coordinates to the side chains of amino acids Asp193, His195, Asp287 (Figures 3 and 4). In HDAC5-SAHA and HDAC5-TSA complexes, the catalytic zinc trihedrally coordinates to the side chains of amino acids Asp178, His180, Asp272. In HDAC6-SAHA and HDAC6-TSA complexes, the catalytic zinc trihedrally coordinates to the side chains of amino acids Asp71, His73, Asp164. In HDAC7-SAHA and HDAC7-TSA complexes, the catalytic zinc trihedrally coordinates to the side chains of amino acids Asp191, His193, Asp285. In HDAC9-SAHA and HDAC9-TSA complexes, the catalytic zinc trihedrally coordinates to the side chains of amino acids Asp188, His190, Asp282. In HDAC10-SAHA and HDAC10-TSA complexes, the catalytic zinc trihedrally coordinates to the side chains of amino acids Asp133, His135, Asp226. Amino acid residues that are involved in binding the catalytic zinc are highly conserved in class II HDACs. In HDAC4-SAHA, HDAC5-SAHA, HDAC6-SAHA and HDAC7-SAHA complexes, SAHA binds the catalytic zinc ion in a bidentate fashion, with its carbonyl and hydroxyl bound to catalytic zinc ion. Whereas, in HDAC9-SAHA and HDAC10-SAHA complexes, SAHA binds the catalytic zinc ion in a monodentate fashion with its carbonyl bound to catalytic zinc ion. Except HDAC5-TSA complex, all of *Homo sapiens *class II HDACs-TSA complexes bind the catalytic zinc ion in a monodentate fashion. The aberrant monodentate coordination of SAHA and TSA in the catalytic domain of *Homo sapiens *class II HDACs -complexes structures originates from the different topology of the active site [21]. In *Homo sapiens *class II HDACs-SAHA complexes, the hydroxyl group of SAHA forms hydrogen bonds with the side chains of two active site histidines, mimicking the interactions of the water molecule in the active site of catalytic domain HDAC7 cocrystal. The hydroxyl group of SAHA replaces the water molecule, which was found in the active site of catalytic domain HDAC7 cocrystal. In *Homo sapiens *class II HDACs-TSA complexes, the hydroxyl group of TSA forms hydrogen bonds with the side chains of two active site histidine and glycine, mimicking the interactions of the water molecule in the active site of catalytic domain HDAC7 cocrystal. The hydroxyl group of SAHA replaces the water molecule, which was found in the active site of catalytic domain HDAC7 cocrystal [22].

**Table 1:** Free binding energy of SAHA and TSA - *Homo sapiens *Class II HDACs Complexes

	Free Binding Energy			
	
	SAHA		TSA	
	
*Homo sapiens *Class II HDACs	APBS (kJ/mol)	AutoDock (kcal/mol)	APBS (kJ/mol)	AutoDock (kcal/mol)
HDAC4	-156.94	-6.16	-156.88	-7.73
HDAC5	-171.77	-7.44	-128.50	-8.19
HDAC6	-213.60	-5.05	-110.62	-5.88
HDAC7	-203.21	-7.42	-144.92	-9.28
HDAC9	-179.29	-6.09	-110.14	-7.85
HDAC10	-263.15	-6.72	-170.79	-7.98

Free binding energy of *Homo sapiens *Class II HDACs-SAHA and TSA complexes obtained under APBS and AutoDock calculations. APBS add intermolecular Colulombic contributions to get the total electrostatic contribution to the binding energy, whereas AutoDock only calculated the sum of the intermolecular energy and the ligand's internal energy.

Each ligand showed different affinities with the class II HDACs; for example, SAHA compound showed the best affinity with the HDAC 5 (-7.44 kcal/mol) based on AutoDock calculation and HDAC10 (-263.15 kJ/mol) based on APBS calculation. Whereas the same compound was found to be rank 2 with HDAC7 (-7.42 kcal/mol) based on AutoDock calculation and HDAC6 (-213.60 kJ/mol) based on APBS calculation, and rank 3 (-6.72 kcal/mol) with HDAC10 (AutoDock) and HDAC7 (-203.21 kJ/mol, APBS). Local free binding energy obtained from AutoDock of *Homo sapiens *class II HDACs complexed with an inhibitor showed that SAHA is a weaker inhibitor of HDACs than TSA. But, global binding energy of *Homo sapiens *class II HDACs and inhibitors obtained from APBS, showed that TSA to be a weaker inhibitor of HDACs than SAHA. There are differences in binding energy calculated with AutoDock and APBS; this is because AutoDock does not calculate columbic contribution from all of atoms in protein like APBS.

The further descriptor analysis and the toxicity prediction (Tables 2 and 3) helped in the identification of the better inhibitor. Drug Score and the Drug-Likeness are the two properties that are important for considering a compound to become a successful drug. TSA had a drug score of 0.37 and drug likeness property score of 1.24, which is higher than those for SAHA with respective scores of 0.35 and -8.87 (Table 2). The molecular weight of SAHA was 264.32 g/mol and that of TSA was 302.37 g/mol (Table 2), between the preferred range of molecular weight for drug likeness property (160-480 g/mol). From Table 3, TSA has red color in tumorigenic property. SAHA was the compound that had the acceptable range (yellow and green colors) for toxicity risk. These values were also taken into account to decide the best inhibitor. Thus, SAHA was the best drug candidate than TSA and also found to possess better global binding affinity score.

**Table 2:** Molecular descriptors value for the SAHA and TSA

Descriptors	SAHA	TSA
LogP	2.47	2.68
Solubility	-3.33	-3.26
Molecular weight	264.32	302.37
TPSA	78.424	69.635
n Atoms	19	22
n ON	5	5
n OHNH	3	2
n Violations	0	0
N Rotatable bonds	8	6
Volume	255.644	293.12
Drug score	0.35	0.37
Drug likeness	-8.87	1.24
Mutagenic	yellow	yellow
Tumorigenic	green	red
Irritant	green	green
Reproductive Effective	green	green

The molecular descriptor values were determined for selected *Homo sapiens *Class II HDACs inhibitor as a drug candidate.

**Table 3:** Toxicity risks of SAHA and TSA

	Toxicity Risks			
	
Inhibitor	Mutagenic	Tumorigenic	Irritant	Reproductive Effective
Suberoyl anilide hydroxamic acid (SAHA)	yellow	green	green	green
Trichostatin A (TSA)	yellow	red	green	green

Prediction results are color coded. Properties with high risks of undesired effects like mutagenicity or a poor intestinal absorption are shown in red. Whereas a green color indicates drug-conform behaviour. From Table 3, TSA was lacking (red color) in one property. SAHA was the compound that had the acceptable range (green color) for tumorigenic, irritant, and reproductive effective.

The ADME-TOX box results showed that the SAHA has an oral bioavailability of more than 70% i.e., good solubility and stability. It acts as a non-substrate and non-inhibitor of P-gp. SAHA does not undergo significant first-pass metabolism. Although the ability to inhibit P-gp is very important in cancer drug characteristics, but SAHA still has the possibility to become a candidate for cervical cancer drug, because cervical tissue is not a P-gp expressing tissue. The predicted LD_50 _values of SAHA in mouse and rat was found to be 1400 mg/kg and 1600 mg/kg respectively when administered orally. SAHA acts as a non-substrate when checked for the P-glycoprotein substrate specificity.

## Conclusion

The three-dimensional models for six class II histone deacetylases (HDAC4, HDAC5, HDAC6, HDAC7, HDAC9, and HDAC10), which were built using homology modeling and validated by bioinformatics techniques and by comparison to an X-ray structures, were docked to SAHA and TSA. Our studies provide the structural view of the catalytic domain of a class II HDAC and reveal for this subclass specific features: (i) a novel zinc binding motif that is likely to be involved in substrate binding and/or protein-protein interactions and may provide a site for modulation of activity, and (ii) a unique active site topology in catalytic activity. SAHA and TSA predicted to inhibit the class II HDACs are effective to all forms HDACs. SAHA was satisfied almost all the properties i.e., binding affinity scores of SAHA in the six class II HDAC enzymes was -156.94 kJ/mol, -171.77 kJ/mol, -213.60 kJ/mol, -203.21 kJ/mol, -179.29 kJ/mol & -263.15 kJ/mol respectively, the Drug Likeness value (1.24) and drug score (0.37) with 70% oral bioavailability and the carbonyl group of these compounds fits well into the active site of the target where the zinc is present. Hence, SAHA could be developed as potential inhibitors of class II HDACs and valuable anti-cancer-agents.

## Competing interests

The authors declare that they have no competing interests.

## References

[B1] Parkin DM The global health burden of infection-associated cancers in the year Int J Cancer 2006 118 3030 3044 10.1002/ijc.21731 16404738

[B2] Lowndes CM Vaccines for cervical cancer Epidemiol Infect 2006 134 1 12, 10.1017/S0950268805005728 16409645 PMC2870376

[B3] Koutsky L Epidemiology of genital human papillomavirus infection Am J Med 1997 102 3 8 10.1016/S0002-9343(97)00177-0 9217656

[B4] Kanodia S Fahey LM Kast WM Mechanisms used by human papillomaviruses to escape the host immune response Current Cancer Drug Targets 2007 7 79 89 10.2174/156800907780006869 17305480

[B5] Kalvatchev Z Rösl F Human papilloma viruses: realities and perspectives Biotechnol Biotechnol Equip 2007 24 137 143

[B6] Walboomers JM Jacobs MV Manos MM Bosch FX Kummer JA Shah KV Snijders PJ Peto J Meijer CJ Muñoz N Human papillomavirus is a necessary cause of invasive cervical cancer worldwide J Pathol 1999 189 12 19 10.1002/(SICI)1096-9896(199909)189:1<12::AID-PATH431>3.0.CO;2-F 10451482

[B7] Bosch FX Manos MM Muñoz N Sherman M Jansen AM Peto J Schiffman MH Moreno V Kurman R Shah KV Prevalence of human papilomavirus in cervical cancer: a worldwide perspective. International biological study on cervical cancer (IBSCC) Study Group J Natl Cancer Inst 1995 87 796 802 10.1093/jnci/87.11.796 7791229

[B8] Thiagalingam S Cheng KH Lee HJ Mineva N Thiagalingam A Ponte JF Histone Deacetylases: Unique Players in Shaping the Epigenetic Histone Code Ann N Y Acad Sci 2003 983 84 100 10.1111/j.1749-6632.2003.tb05964.x 12724214

[B9] de Ruijter AJ van Gennip AH Caron HN Kemp S van Kuilenburg AB Histone deacetylases (HDACs): characterization of the classical HDAC family Biochem J 2003 370 737 49 10.1042/BJ20021321 12429021 PMC1223209

[B10] Subha K Kumar GR Assessment for the identification of better HDAC inhibitor class through binding energy calculations and descriptor analysis Bioinformation 2008 3 218 222 19255637 10.6026/97320630003218 PMC2646192

[B11] Finzer P Kuntzen C Soto U zur Hausen H Rösl F Inhibitors of histone deacetylase arrest cell cycle and induce apoptosis in cervical carcinoma cells circumventing human papillomavirus oncogene expression Oncogene 2001 20 4768 76 10.1038/sj.onc.1204652 11521189

[B12] Finzer P Ventz R Kuntzen C Seibert N Soto U Rösl F Growth arrest of HPV-positive cells after histone deacetylase inhibition is independent of E6/E7 oncogene expression Virology 2002 304 265 73 10.1006/viro.2002.1667 12504567

[B13] Finzer P Krueger A Stöhr M Brenner D Soto U Kuntzen C Krammer PH Rösl F HDAC inhibitors trigger apoptosis in HPV-positive cells by inducing the E2F-p73 pathway Oncogene 2004 23 4807 17 10.1038/sj.onc.1207620 15077164

[B14] Acharya MR Sparreboom A Sausville EA Conley BA Doroshow JH Venitz J Figg WD Interspecies differences in plasma protein binding of MS-275, a novel histone deacetylase inhibitor Cancer Chemother Pharmacol 2006 57 275 81 10.1007/s00280-005-0058-8 16028097

[B15] Thompson JD Higgins DG Gibson TJ CLUSTAL W: improving the sensitivity of progressive multiple sequence alignment through sequence weighting, position-specific gap penalties and weight matrix choice Nucleic Acids Res 1994 22 4673 80 10.1093/nar/22.22.4673 7984417 PMC308517

[B16] Martí-Renom MA Stuart AC Fiser A Sánchez R Melo F Sali A Comparative protein structure modeling of genes and genomes Annu Rev Biophys Biomol Struct 2000 29 291 325 10.1146/annurev.biophys.29.1.291 10940251

[B17] Hildebrand A Remmert M Biegert A Söding J Fast and accurate automatic structure prediction with HHpred Proteins 2009 77 Suppl 9 128 32 19626712 10.1002/prot.22499

[B18] Dolinsky TJ Czodrowski P Li H Nielsen JE Jensen JH Klebe G Baker NA PDB2PQR: expanding and upgrading automated preparation of biomolecular structures for molecular simulations Nucleic Acids Res 2007 35 W522 5 10.1093/nar/gkm276 17488841 PMC1933214

[B19] Lipinski CA Lombardo F Dominy BW Feeney PJ Experimental and computational approaches to estimate solubility and permeability in drug discovery and development settings Adv Drug Deliv Rev 2001 46 3 26 10.1016/S0169-409X(00)00129-0 11259830

[B20] Willard L Ranjan A Zhang H Monzavi H Boyko RF Sykes BD Wishart DS VADAR: a web server for quantitative evaluation of protein structure quality Nucleic Acids Res 2003 31 3316 9 10.1093/nar/gkg565 12824316 PMC168972

[B21] Bottomley MJ Lo Surdo P Di Giovine P Cirillo A Scarpelli R Ferrigno F Jones P Neddermann P De Francesco R Steinkühler C Gallinari P Carfí A Structural and functional analysis of the human HDAC4 catalytic domain reveals a regulatory structural zinc-binding domain J Biol Chem 2008 283 26694 704 10.1074/jbc.M803514200 18614528 PMC3258910

[B22] Schuetz A Min J Allali-Hassani A Schapira M Shuen M Loppnau P Mazitschek R Kwiatkowski NP Lewis TA Maglathin RL McLean TH Bochkarev A Plotnikov AN Vedadi M Arrowsmith CH Human HDAC7 harbors a class IIa histone deacetylase-specific zinc binding motif and cryptic deacetylase activity J Biol Chem 2008 283 11355 63 10.1074/jbc.M707362200 18285338 PMC2431080

[B23] Wang DF Helquist P Wiech NL Wiest O Toward selective histone deacetylase inhibitor design: homology modeling, docking studies, and molecular dynamics simulations of human class I histone deacetylases J Med Chem 2005 48 6936 47 10.1021/jm0505011 16250652

